# Complete genomes of *Limosilactobacillus portuensis* and *Limosilactobacillus vaginalis* isolated from the urine of postmenopausal women

**DOI:** 10.1128/MRA.00883-23

**Published:** 2023-11-29

**Authors:** Vivian H. Nguyen, Belle M. Sharon, Brianna M. Shipman, Philippe E. Zimmern, Nicole J. De Nisco

**Affiliations:** 1 Department of Biological Sciences, The University of Texas at Dallas, Richardson, Texas, USA; 2 Department of Urology, The University of Texas at Southwestern Medical Center, Dallas, Texas, USA; Wellesley College, Wellesley, Massachusetts, USA

**Keywords:** human microbiome, urinary tract infection, genomes, *Limosilactobacillus*, lactic acid bacteria

## Abstract

There is frequent evidence that *Limosilactobacillus vaginalis* colonizes female genitourinary tracts but few reports of *Limosilactobacillus portuensis*. Their role in urinary tract infection (UTI) is unclear. We present the first complete genome of *L. portuensis* and a complete genome of *L. vaginalis* isolated from postmenopausal women with varying UTI histories.

## ANNOUNCEMENT

Certain species of *Lactobacillus* and *Limosilactobacillus* are believed to play a protective role in the female genitourinary tract (1, 2). However, the roles of *Limosilactobacillus portuensis* and *Limosilactobacillus vaginalis* are unclear given the phenotypic heterogeneity of urinary lactobacilli and variability in abundance between patients (3). Therefore, the genomes of these understudied species must be analyzed to better understand their role in the urogenital environment and UTI susceptibility. Complete genomes of *Limosilactobacillus* species are necessary to identify genetic elements that are associated with disease states and begin to determine how they may contribute to host colonization. As part of an institutional review board-approved study (STU 032016-006 and MR 17-120), we cultivated, sequenced, and assembled the complete, closed genomes of two *Limosilactobacillus strains* isolated from the urine of postmenopausal with and without histories of recurrent UTI collected at the University of Texas Southwestern Medical Center in Dallas, Texas.

Clean-catch midstream urine was obtained from one postmenopausal women with a history of recurrent UTI history who was currently asymptomatic and one postmenopausal women with no UTI history (2). Urine was plated on De Man, Rogosa, and Sharpe agar and incubated for 72 h at 35°C under microaerophilic conditions using BD GasPak EZ CampyPouch systems and well-isolated colonies were restruck prior to cryostorage. Initial species identification was performed using Sanger sequencing of the 16S rRNA gene and BLASTn was queried against the NCBI 16S ribosomal RNA (Bacteria and Archaea) database as previously described (2). *L. portuensis* was isolated from the woman with recurrent UTI history, and *L. vaginalis* was isolated from the woman with No UTI history (Table 1). Single-colony isolates were grown in MRS broth under microaerophilic conditions for genomic DNA extraction using the Qiagen DNeasy blood and tissue kit, and DNA quality was assessed using gel electrophoresis, A260/280, and Qubit fluorometer (4).

**TABLE 1:** Strain name, accession numbers, assembly parameters, and isolate characteristics of two *Limosilactobacillus* strains

Strain	Species	Host health state	BioSample accession no.	SRA accession no^*a*^	No. of raw reads	No. of trimmed reads	ONT read N50 (bp)	Read depth (×)	GenBank accession No.	Type of contig (circular)	Total length (bp)	GC content (%)	No. of CDs^*b*^
LP1276_C155	*Limosilactobacillus portuensis*	UTI history	SAMN33053616	SRR25546982 (I)	5,987,450	5,916,322 (I)	4,666	396	CP117296	Chromosome	1,928,671	40.3	1,967
				SRR25546981 (O)	309,028	308,216 (O)		408	CP117297	Plasmid	62,042	40.6	71
LV1494_C165	*Limosilactobacillus vaginalis*	No UTI history	SAMN33053617	SRR25546980 (I)	3,844,010	3,798,743 (I)	1,717	293	CP117295	Chromosome	1,837,310	40.6	1,834
				SRR25546979 (O)	822,661	821,302 (O)		594					

^*a*^ (O), ONT; (I), Ilumina.

^*b*^ CDs, coding sequences.

Illumina library preparation was performed using the Nextera DNA Flex kit and 2 × 150 bp paired-end reads were generated using a NextSeq500. CLC Genomics Workbench v12.0.3 was used to assess read quality and to trim reads with Phred score <20 and read length <15 bp. The same genomic DNA was used for Oxford Nanopore libraries preparation via the ligation sequencing kit (SQK-LSK109) and barcode expansion kit 1–12 (EXP-NBD104) and no DNA shearing or size-selection was performed. Libraries were sequenced on a MinION platform using R9 FLO-MIN106 flow cells with ONT MinKNOW software for live fast base calling, demultiplexing, and barcode trimming. NanoStat v1.2.0 was used for quality assessment and NanoFilt v2.6.0 for trimming. Reads with length >200 bp and Phred score >7 were retained (5). Read metrics are found in Table 1.

Hybrid assemblies of Illumina and ONT reads were created using the default mode of Unicycler v0.4.8 (6), SPAdes v3.13.0 (7), Racon v1.4.10 (8), and Pilon v1.23 (9). Genomes were rotated to the start of the dnaA or repA gene. QUAST v5.0.2 was used to evaluate assembly quality (10). Bandage v0.8.1 and BUSCO v1 were used to visualize assembly graphs and assess genome completeness using ortholog set on the gVolante v.12 server (11–13). Genomes were annotated using the NCBI Prokaryotic Genome Annotation Pipeline v6.4 (14). GC content and coding sequences were determined using Geneious Prime v2020.0.5 (Table 1). Analysis parameters were default except where otherwise noted. The closed *L. portuensis* genome harbored a chromosome of ~1.93 Mb and a plasmid of ~62 kb, while the closed *L. vaginalis* genome was only comprised of a ~1.84 Mb chromosome (Table 1).

## Data Availability

This project has been deposited in GenBank under the BioProject PRJNA930673. Table 1 displays BioSample and SRA accession numbers associated with each isolate.
